# Predicting overall survival in diffuse glioma from the presurgical connectome

**DOI:** 10.1038/s41598-022-22387-7

**Published:** 2022-11-05

**Authors:** Shelli R. Kesler, Rebecca A. Harrison, Vikram Rao, Hannah Dyson, Melissa Petersen, Sarah Prinsloo

**Affiliations:** 1 Department of Adult Health, School of Nursing, University of Texas at Austin, 1710 Red River St, Austin, TX D010078712 USA; 2 Department of Diagnostic Medicine, Dell School of Medicine, University of Texas at Austin, Austin, TX USA; 3 Department of Oncology, Dell School of Medicine, University of Texas at Austin, Austin, TX USA; 4 Division of Neurology, University of British Columbia, Vancouver, BC Canada; 5 Department of Internal Medicine, University of North Texas Health Science Center, Fort Worth, TX USA; 6 Department of Family Medicine, University of North Texas Health Science Center, Fort Worth, TX USA; 7 Department of Neurosurgery, MD Anderson Cancer Center, Houston, TX USA

**Keywords:** Cancer, Computational neuroscience, Predictive markers

## Abstract

Diffuse gliomas are incurable brain tumors, yet there is significant heterogeneity in patient survival. Advanced computational techniques such as radiomics show potential for presurgical prediction of survival and other outcomes from neuroimaging. However, these techniques ignore non-lesioned brain features that could be essential for improving prediction accuracy. Gray matter covariance network (connectome) features were retrospectively identified from the T1-weighted MRIs of 305 adult patients diagnosed with diffuse glioma. These features were entered into a Cox proportional hazards model to predict overall survival with 10-folds cross-validation. The mean time-dependent area under the curve (AUC) of the connectome model was compared with the mean AUCs of clinical and radiomic models using a pairwise t-test with Bonferroni correction. One clinical model included only features that are known presurgery (clinical) and another included an advantaged set of features that are not typically known presurgery (clinical +). The median survival time for all patients was 134.2 months. The connectome model (AUC 0.88 ± 0.01) demonstrated superior performance (P < 0.001, corrected) compared to the clinical (AUC 0.61 ± 0.02), clinical + (AUC 0.79 ± 0.01) and radiomic models (AUC 0.75 ± 0.02). These findings indicate that the connectome is a feasible and reliable early biomarker for predicting survival in patients with diffuse glioma. Connectome and other whole-brain models could be valuable tools for precision medicine by informing patient risk stratification and treatment decision-making.

## Introduction

Diffuse gliomas are the most common malignant primary brain tumor. While our understanding of the biologic foundations of these cancers has grown in recent years, all diffuse gliomas are considered incurable at this time. However, median survival broadly ranges up to approximately 15 years, varying within histologic and molecular subgroups^1^. Predicting survival from baseline characteristics is especially useful in risk stratification and planning individualized therapy. Patients with favorable prognostic indicators at baseline may potentially be spared unnecessarily aggressive treatments^2^. Improving preoperative diagnostics may also facilitate the development of window-of-opportunity clinical trials in neuro-oncology, where the difficulty of obtaining diagnostic biomarkers hinders the development of these therapeutic studies.

Persistent attempts to refine prognostic measures have been made based on histology and extent of surgical resection. Malignant astrocytomas are associated with lower progression-free and overall survival compared to oligodendrogliomas and oligoastrocytomas^3^. Gross total tumor resection is not always possible but is associated with improved survival outcome^4^. However, these variables are not typically known in advance of surgery. Tumor genotyping represents one of the most promising methods for risk stratification. For example, patients with isocitrate dehydrogenase wild-type tumors tend to have significantly lower survival rates compared to patients with mutant tumors^5^. However, genomic and epigenomic tumor profiling requires neurosurgery and can be limited by the volume and quality of the tissue sample, available expertise, and cost. As such, current clinical practice does not allow for comprehensive molecular assessment on all patients. Further, some studies have found that isocitrate dehydrogenase status is poorly associated with long-term survival in high grade tumors^6^.

Neuroimaging is standard of care prior to neurosurgery for brain tumors and represents a noninvasive, early biomarker for risk stratification. Advanced computational imaging techniques, such as radiomics, have demonstrated potential for predicting survival in patients with diffuse glioma from presurgical neuroimaging^7^. However, radiomic methods utilize only imaging features of the tumor itself, ignoring the rest of the brain. Our group and others have demonstrated that diffuse gliomas are associated with wide-spread brain network disruption^8–10^. These findings reflect the fact that gliomas are not focal tumors, but malignancies that propagate throughout the brain. In fact, this dissemination is one of the reasons these tumors tend to recur and remain difficult to treat. Therefore, a focus solely on tumor characteristics likely neglects important prognostic information. Accordingly, recent evidence suggests that changes in whole-brain networks (connectomics) may serve as indicators of survival^10–12^. However, connectome models have not been compared to radiomic models in prior studies. We developed and cross-validated multivariable models for predicting overall survival in diffuse glioma.

Few if any studies have evaluated T1-weighted MRI (T1w MRI) based connectome models of glioma survival. T1w MRI is routinely acquired presurgically as part of standard of care for patients with brain tumors. T1w MRI is used ubiquitously in neuroimaging research, including our own, to measure brain volumes^13–18^. There exist coordinated variations in gray matter volumes that make connectome construction possible^19^. These structural covariance networks are highly heritable^20^ and are believed to reflect underlying axonal connections as well as common neurodevelopmental and neuroplastic processes involved in the formation of functional neural communities^21–23^. Accordingly, our group and others have shown that gray matter connectomes are consistent with diffusion tensor imaging (DTI) and functional MRI (fMRI) derived connectomes^24,25^ and are highly reproducible and reliable^26^. We hypothesized that a T1w MRI connectome model would outperform both clinical and radiomic models in predicting glioma survival.

## Methods

### Participants

We retrospectively identified adult (age 18 or older) patients with histopathologically confirmed World Health Organization grade II–IV gliomas who were newly diagnosed and first treated at MD Anderson Cancer Center. A total of 305 patients met these criteria and had an available pre-surgical, T1w MRI acquired at 3 T. Patients were treated during the years of 1996–2020. MRI, demographic, and other clinical data were extracted from the electronic medical record. This study, including a waiver of written informed consent, was approved by the MD Anderson Cancer Center Institutional Review Board (protocol# 2021-0236). The study was conducted in accordance with the Declaration of Helsinki.

### Connectome predictors

Gray matter volumes were segmented from T1w MRI with Voxel-Based Morphometry v8 and Statistical Parametric Mapping v12 (Wellcome Trust Centre for Neuroimaging, London, UK). We employed Diffeomorphic Anatomical Registration Through Exponentiated Lie Algebra, which uses a large deformation framework to preserve topology and employs customized, sample-specific templates resulting in superior image registration, even in lesioned brains, compared to other automated methods^27^. Successful normalization was confirmed using visual and quantitative quality assurance methods^9^.

A connectome was constructed for each patient from gray matter covariance networks using a similarity-based extraction method^28^. Specifically, network nodes were defined as 3 × 3 × 3 voxel cubes spanning the entire gray matter volume (i.e. 54 gray matter values per cube). A correlation matrix was calculated across all pairs of nodes and binarized based on a threshold estimated from a random network with false discovery rate^28,29^. We ensured that no binarized matrices were disconnected (i.e., had isolated nodes). We then applied graph theoretical analysis using the bNets Toolbox v2.2 (Brain Health Neuroscience Lab, Austin, TX)^30^ and Brain Connectivity Toolbox v2019-03-03^31^ to calculate local efficiency^32^ for each connectome node. Local efficiency is consistently observed to be affected in patients with diffuse glioma^9,33–35^. We also computed total brain volume, connectome size (number of nodes) and degree (number of nodal connections) as these can bias connectome measurements. Connectome size naturally varies across individuals, so gray matter volumes were collapsed across 90 cortical and subcortical regions^36^ to facilitate analyses^9^. The 90 efficiency values, brain volume, size and degree provided a total of 93 connectome predictors.

### Radiomic predictors

Tumor segmentation was performed on the T1w MRI volume in the axial plane using 3D Slicer v4.11 (Slicer Community, Cambridge, MA)^37^. Specifically, two seed regions were created manually for a limited number of slices representing superior, midpoint and inferior aspects of the tumor. For each slice, one seed was set in the tumor area and the second was placed in the non-tumor area. The Fast GrowCut method was then employed to efficiently interpolate a full segmentation of the entire volume from the seed regions. Two expert raters reviewed the segmentations for accuracy.

Radiomic features were extracted from the tumor segmentation using PyRadiomics v3.01 (PyRadiomics Community, Boston, MA), a Python-based, open-source software package that has a 3D Slicer integration. PyRadiomics implements hard-coded algorithms for image processing and feature definition to improve the standardization and reproducibility of radiomics analyses^38^. Another advantage of PyRadiomics is the ability to extract radiomic features from a single T1w MRI volume, making this approach the most comparable to our connectome model. We obtained at total of 107 PyRadiomics predictors including 14 shape features, 18 intensity features and 75 texture features.

### Clinical predictors

Available clinical variables included primary tumor location (1 = occipital, 2 = parietal,3 = temporal,4 = frontal) and hemispheric laterality (left = 1, right = 0), multifocal tumor (yes = 1, no = 0), tumor grade (2,3,4), histology (1 = astrocytoma, 2 = oligodendroglioma; 3 = oligoastrocytoma), extent of surgical resection (1 = gross total, 2 = subtotal, 3 = biopsy), patient age at diagnosis in years, and biological sex (1 = male, 0 = female) for a total of 8 predictors. Other known predictors of glioma survival such as tumor genotype and Karnofsky performance status were not available for most participants. It is important to note that tumor grade, histology and extent of resection are not typically known presurgery, so this represented an advantaged model compared to the neuroimaging models. We therefore also examined a truly presurgical clinical model that included only primary tumor location, laterality, multifocality, age at diagnosis, and biological sex (5 predictors).

### Statistical analysis

We fit a Cox proportional hazards model to predict overall survival time in months for each of four models, one with connectome covariates, one with presurgical only clinical covariates (clinical model), one with the advantaged clinical covariates (clinical + model), and a fourth with radiomics covariates. Given the large number of covariates, we implemented regularized regression^39^ and 10-folds cross-validation^40^ to decrease model complexity and reduce overfitting. Specifically, the N = 305 datasets were randomly shuffled and then split into 10 subsets (i.e., folds). For each of the 10 cross-validation loops, a Cox proportional hazards model with a ridge penalty was trained on the data from 9 of the folds and then tested on the left-out fold such that every fold was tested once.

Model performance for predicting survival time in the test fold was measured using the time-dependent AUC of the receiver operating characteristic (ROC). In this case, the AUC was the integral of AUC on the range of survival time from 0 to maximum, weighted by the estimated probability density of the time-to-event outcome. In other words, this measurement takes into account the time-dependent nature of the parameters and the impact of censoring^41^. The AUC was averaged across the 10 cross-validation loops. We compared the mean AUC from each of these models using a pairwise t-test with Bonferroni correction for multiple comparisons.

## Results

**Figure 1:**
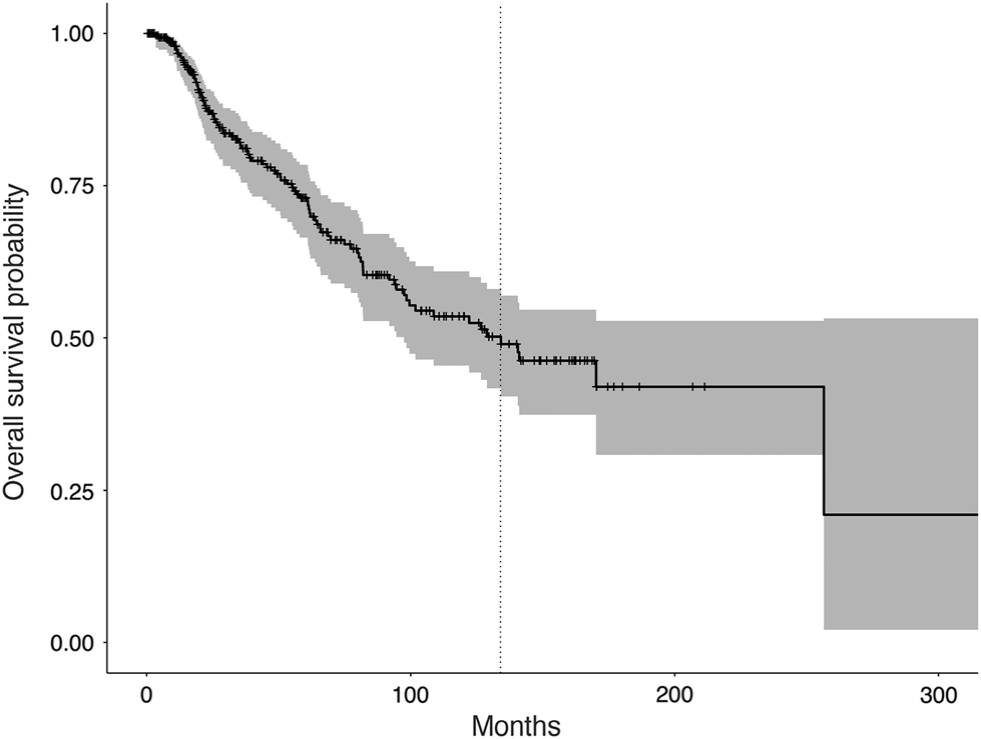
Kaplan–Meier plot of overall survival probability. Dotted vertical line indicates median survival time of 134.2 months.

All 305 patients identified for analysis had complete data. Patient characteristics are outlined in Table 1. Age at diagnosis ranged from 18 to 82 years and most patients were male. Most patients had left frontal, high grade tumors and received gross total resection. Median survival time was 134.2 months, ranging from 0.36 to 334.8 months (Fig. 1).

**Table 1:** Patient characteristics N = 305.

	N (%)
Age at diagnosis	43.96 ± 14.8 years
**Biological sex**	
Male	180 (59.0%)
Female	125 (41.0%)
**Histologic phenotype**	
Astrocytoma	221 (72.4%)
Oligodendroglioma	69 (22.6%)
Oligoastrocytoma	15 (5.0%)
**Histologic grade**	
Grade II	123 (40.3%)
Grade III	79 (25.9%)
Grade IV	103 (33.8%)
**Tumor laterality**	
Right	86 (28.2%)
Left	219 (71.8%)
**Primary tumor location**	
Frontal	154 (50.5%)
Temporal	105 (34.4%)
Parietal	44 (14.4%)
Occipital	2 (0.7%)
**Extent of resection**	
Gross total resection	136 (44.6%)
Subtotal resection	108 (35.4%)
Biopsy	61 (20.0%)

**Figure 2:**
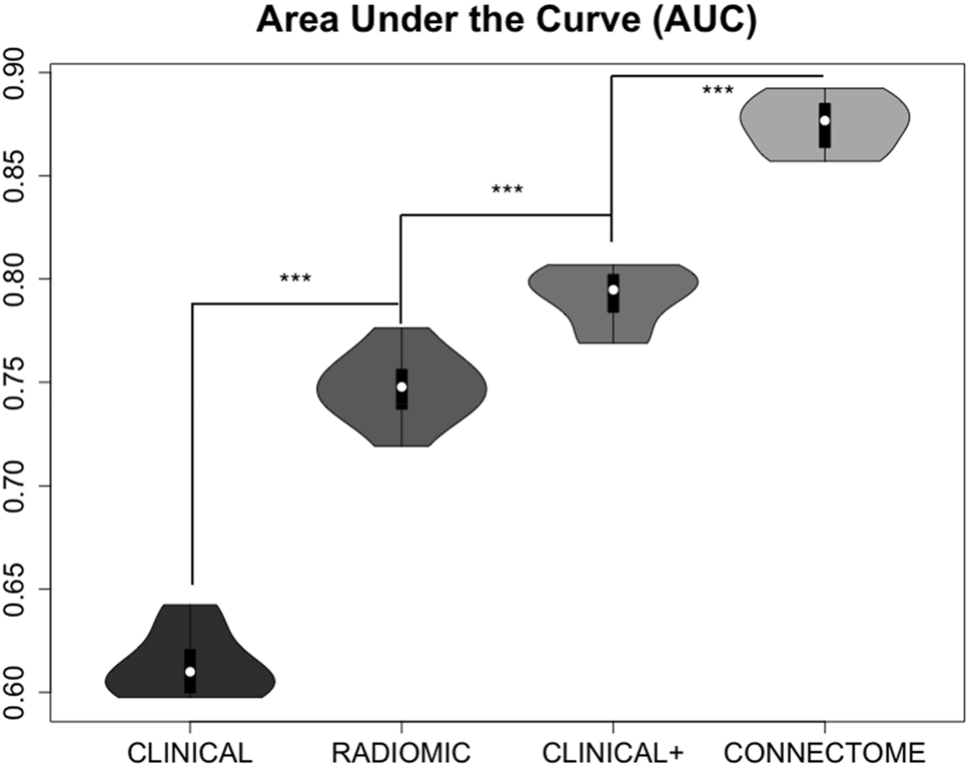
Violin plots of AUC values across 10-folds cross-validation. The connectome model (AUC 0.88 ± 0.01) demonstrated superior performance compared to the clinical (AUC 0.61 ± 0.02), clinical + (AUC 0.79 ± 0.01) and radiomic models (AUC 0.75 ± 0.02) in predicting overall survival. The clinical + and radiomics models outperformed the clinical model and the clinical + model outperformed the radiomics model. *** P < 0.001, corrected.

As shown in Fig. 2, the connectome model (AUC 0.88 ± 0.01) demonstrated superior performance (P < 0.001, corrected) compared to the clinical (AUC 0.61 ± 0.02), clinical + (AUC 0.79 ± 0.01) and radiomic models (AUC 0.75 ± 0.02) in predicting overall survival. The clinical + and radiomics models outperformed the clinical model (P < 0.001, corrected) and the clinical + model outperformed the radiomics model (P < 0.001, corrected).

**Figure 3:**
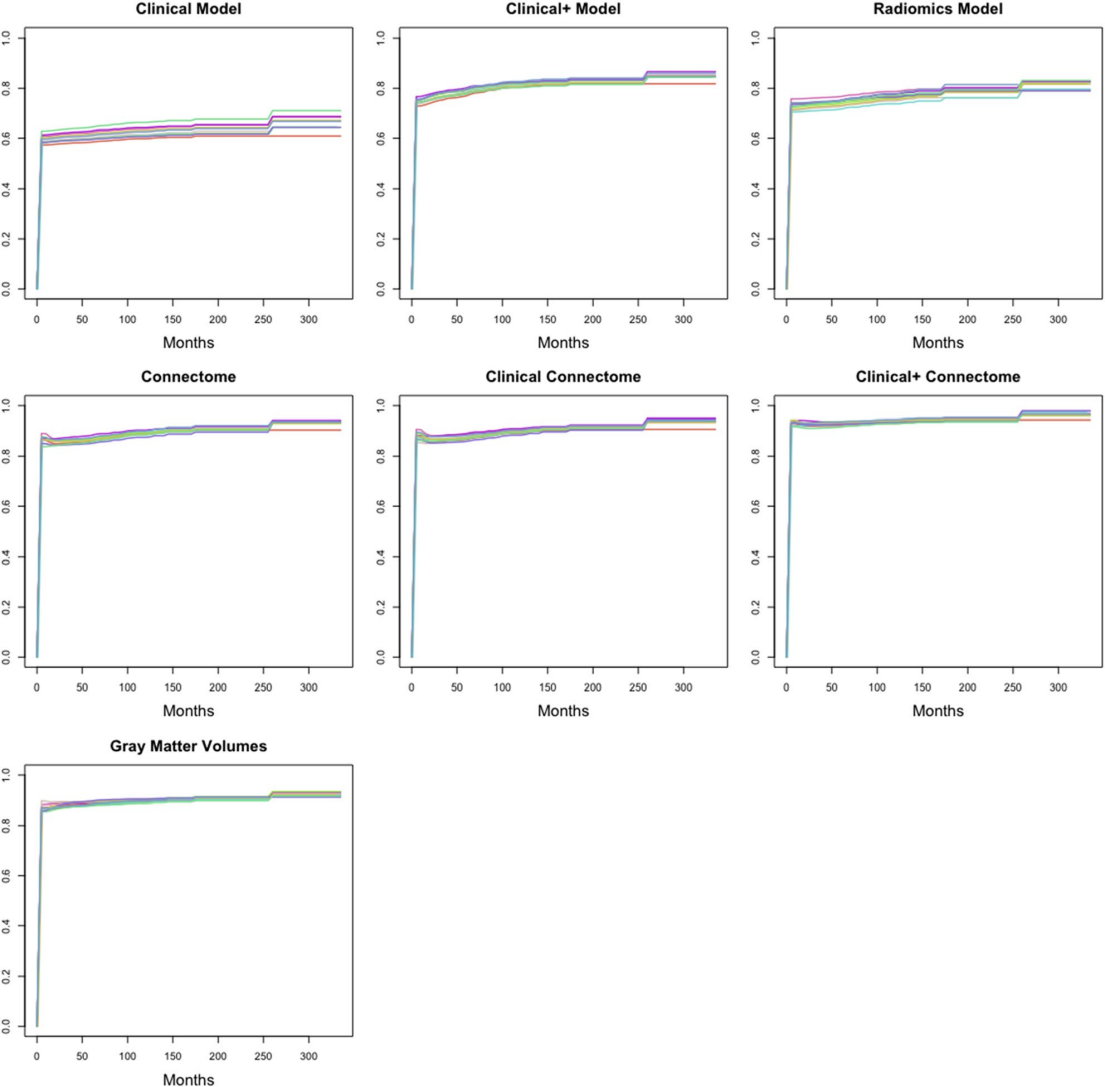
Receiver operating characteristic (ROC) curves. The ROC curve for each of the 10 cross-validation folds are shown here for the primary (clinical, clinical +, radiomics, connectome) and post-hoc (clinical connectome, clinical + connectome, gray matter volumes) models predicting overall glioma survival.

To further interpret our findings, we conducted three post-hoc models, one that combined clinical and connectome features, one that combined clinical + and connectome features, and one that included gray matter volumes extracted from the same 90 regions of interest that were applied to the connectome efficiencies. Regional volumes were measured from the segmented gray matter image for each participant using the fslstats tool in FMRIB Software Library v6.0 (FMRIB, Oxford, UK)^42^. The connectome/clinical model demonstrated a mean AUC of 0.88 ± 0.01, the connectome/clinical + model demonstrated a mean AUC of 0.93 ± 0.01), and the gray matter volumes model demonstrated a mean AUC of 0.88 ± 0.01. The connectome/clinical + model significantly outperformed the connectome model (P < 0.001) and the gray matter volumes model was significantly better than the clinical + model (P < 0.001). ROC curves for each fold of each model are presented in Fig. 3.

The emphasis of machine learning models such as these is on generalizable models, and they are not designed for inference. In other words, the goal of this and similar studies is not to determine specific factors that predict outcome, but to develop reproducible models that perform well across samples. Cross-validation results in a different Cox proportional hazards fit within each fold and thus it is not possible to precisely determine the coefficients or significance values for the covariates. However, given that this is the first gray matter connectome model of glioma survival, examination of individual predictors may provide important insights for future studies. Therefore, predictors with a mean p value of 0.05 or less across the 10 cross-validation loops are listed for each model in Table 2. Coefficients and p values for every predictor, for every fold of every model are provided in the Supplementary Data.

**Table 2:** Model predictors with a mean p value of 0.05 or less across cross-validation loops.

Clinical	None
Clinical +	Tumor grade (0.98), tumor lobe (− 0.24)
Radiomic	None
Connectome^a^	Left inferior triangularis (− 124), right rolandic operculum (− 97), right anterior cingulum (− 135), right hippocampus (− 96), right calcarine (− 87), left cuneus (− 80), right superior occipital (90), left paracentral lobule (− 94), right putamen (− 86), right pallidum (58), right thalamus (124), left superior temporal pole (97)
Connectome/clinical	Gender (− 1.1), brain volume (0.005), left inferior triangularis (− 107), right rolandic operculum (− 110), right anterior cingulum (− 132), right hippocampus (− 98), right calcarine (− 85), left paracentral lobule (− 86), right thalamus (123), left superior temporal pole (104)
Connectome/Clinical +	Tumor grade (− 1.5), left inferior triangularis (25), right inferior orbitofrontal (50), right rolandic operculum (− 44), right anterior cingulate (72), right superior occipital (107), left paracentral lobule (60), right thalamus (− 33), left superior temporal pole (54), right superior temporal pole (83)
Gray matter volume^a^	Left cuneus (0.002), left inferior triangularis (0.001), right inferior triangularis (− 0.003), left superior parietal (− 0.002), left inferior temporal (0.001)

The mean unstandardized coefficient is shown in parenthesis.

^a^See Supplemental Fig. S1 for visualization of predictive regions.

## Discussion

We evaluated the accuracy of connectome features derived from presurgical T1w MRI to predict overall survival in patients with diffuse glioma. We compared the connectome model to a presurgical clinical model, an advantaged clinical model that included both pre- and post-surgically known features, and a model with radiomic features, also derived from the presurgical T1w MRI. As hypothesized, we found that the connectome model’s performance was superior to all other models, having the highest cross-validated mean AUC. The differences in AUCs between the models were highly significant (P < 0.001), even after controlling for multiple comparisons. Thus, although three of the four models showed strong performance, the connectome model was superior beyond chance. These findings illustrate the valuable prognostic information contained within the entire brain, not limited to tumor characteristics.

Notably, our connectome model demonstrated equal or better predictive accuracy than the radiomics model within our own sample and in comparison to previous studies, including those that used manual tumor segmentation and multiple imaging sequences^43,44^. Manual tumor segmentation is time consuming, requiring one or more expert raters. Even semi-automated methods such as the one we employed here require expert raters, which can reduce reliability and reproducibility. In comparison, extracting connectome features is completely automated and our open-source connectome software tools for doing so are publicly available^45^. The various imaging sequences used in prior radiomics studies require additional processing and are not consistently available. Alternatively, our connectome features were derived from a single, standard of care, T1w MRI volume.

No prior studies have evaluated T1w MRI -based connectome models of glioma survival. Our T1w MRI connectome model was superior to previously reported fMRI and DTI based connectome models in predicting glioma survival, which have demonstrated AUCs or accuracies of 0.75–0.81^11,12,46^. T1w MRI has distinct advantages compared to DTI in terms of reliability in constructing brain networks and standardizing pulse sequences across scanner types^47,48^. Unlike fMRI, T1w MRI is more routinely prescribed as presurgical, standard of care. T1w MRI has greater resistance to artifact over other imaging modalities and requires less resource to preprocess and analyze than other acquisitions^47^.

Tumor cells are difficult to differentiate from gray matter in T1w MRI and are intermixed with normally functioning tissue within the tumor^49^. Preservation of functional cells within tumor boundaries has been associated with improved survival^50^ and pathologic neural networks formed by glioma cells promote tumor progression through growth factor release^51^. Therefore, all voxels within the tumor boundary are important to include because the relationships of those voxel in the network will theoretically reflect the pathology of the tumor and be important for predicting outcomes. This constitutes a true *whole* brain approach, rather than a tumor only or non-tumor only approach.

The connectome model outperformed the clinical model but also the advantaged clinical model, which included tumor grade and histology features that are not typically known presurgery. The superior accuracy of the connectome model likely reflects the whole brain network’s incorporation of variables that are both intrinsic and extrinsic to the tumor. The combination of presurgically known clinical variables with connectome predictors did not improve model performance suggesting that the connectome incorporates some or all of these clinical features. Previous studies have provided evidence that connectome metrics reflect important molecular properties of the tumor. Our group and others have demonstrated that isocitrate dehydrogenase wild-type tumors are associated with significantly greater connectome disruption compared to mutant tumors^8,9^. We also showed that connectome properties can predict isocitrate dehydrogenase tumor status with high accuracy^52^. Connectome organization has been associated with other prognostic factors such as tumor grade, Karnofsky performance status, cognitive function, age, socioeconomic status, psychological function, and biological sex^10,53–57^. However, the addition of clinical + and connectome features resulted in a superior model indicating that the connectome may not reflect certain postsurgically known variables such as tumor grade.

Local efficiencies in multiple brain regions were associated with overall survival in the connectome models. While most patients had left-hemispheric tumors, nearly twice as many right-sided brain regions were predictive of overall survival. Additionally, the predictive regions were not limited to the frontal areas where most tumors were located, but were spread throughout frontal, subcortical, temporal, and postcentral midline areas. These findings provide further evidence that gliomas have broad ranging effects within the brain network. Widespread effects may reflect propagation of pathology through tumor-induced neural networks^51,58^ or neuroplastic adaptation of the brain to the tumor. Both mechanisms would depend on tumor type, with more aggressive tumors demonstrating greater disease propagation and less neuroplastic adaptation^59^. Our results indicated that local efficiencies were largely inversely related to survival. Higher efficiency reflects a greater number of direct connections between brain regions^32^, which may facilitate propagation of pathology. Local efficiency is also directly related to error tolerance^60,61^ and therefore, the regions identified by our connectome model may be particularly vulnerable to disseminated tumor effects, resulting in decreased survival.

The performance of the gray matter volume model was better than the clinical + and radiomics models and equal to the connectome model. These findings again suggest that the use of whole brain data for predicting glioma outcome is superior to tumor-limited data, but connectivity between regions may not be the critical factor. However, regression considers the additive relationship between predictors based on the general linear model and this multivariate space is how brain connectivity is defined. Thus, the gray matter volume model may not be entirely devoid of connectivity information. Despite equivalent model performance, the connectome data provide greater insight regarding potential mechanisms of brain network disruption, as noted above. However, volume data are easier to calculate compared to connectome properties and should be explored further in terms of predicting glioma outcomes.

This was a retrospective study with a limited number of available clinical predictors. Our clinical, connectome, and radiomic models require further validation in an independent dataset to verify their value in predicting patient survival. We used connectome features based on prior literature but recognize that alternative metrics may yield different results. Studies with larger sample sizes are required to examine and compare additional connectome features. We limited our sample to 3 T field strength imaging which may not be available in all clinical settings. An additional goal of further research would be to compare these results with other advanced imaging modalities, such as DTI and fMRI, as well as multi-sequence-based radiomics. Future studies with larger samples should further evaluate the accuracy of models that combine neuroimaging and clinical features.

In summary, we provide promising evidence that overall survival in patients with glioma can be accurately predicted by presurgical connectome features. Our results also emphasize the prognostic information that can be obtained from standard T1w MRI sequences to support precision medicine applications. The connectome and gray matter volumes models significantly outperformed clinical and radiomic models. This work suggests that whole-brain data are valuable and easily attainable biomarkers that can provide an early understanding of glioma trajectory. Data regarding patient prognosis at time of diagnosis influences multiple aspects of patient care, including the nature and aggressiveness of anti-cancer treatment, counseling of patients and family members, and goals of care planning. With further validation, our work may ultimately provide a novel method to refine the biologic stratification of patients in clinical trials and clinical practice.

## Supplementary Information

Supplementary Information 1.

Supplementary Information 2.

## Data Availability

All data relevant to the study are included in the article. The original MRI data underlying this article cannot be shared publicly due to data protection regulation, but connectome matrices are available upon request to the corresponding author (srkesler@austin.utexas.edu). All preprocessing and analysis codes are available at https://github.com/srkesler.
