# Supplementary material for: Predicting overall survival in diffuse glioma from the presurgical connectome
Source: Sci Rep. 2022 Nov 5;12:18783. doi: 10.1038/s41598-022-22387-7 (PMC9637134; doi:10.1038/s41598-022-22387-7)
Supplement: Supplementary file 2 — Supplementary Information 2. [file 41598_2022_22387_MOESM2_ESM.docx]

Supplemental Figure 1. Significant Brain Regions for Connectome and Gray Matter Volume Models.


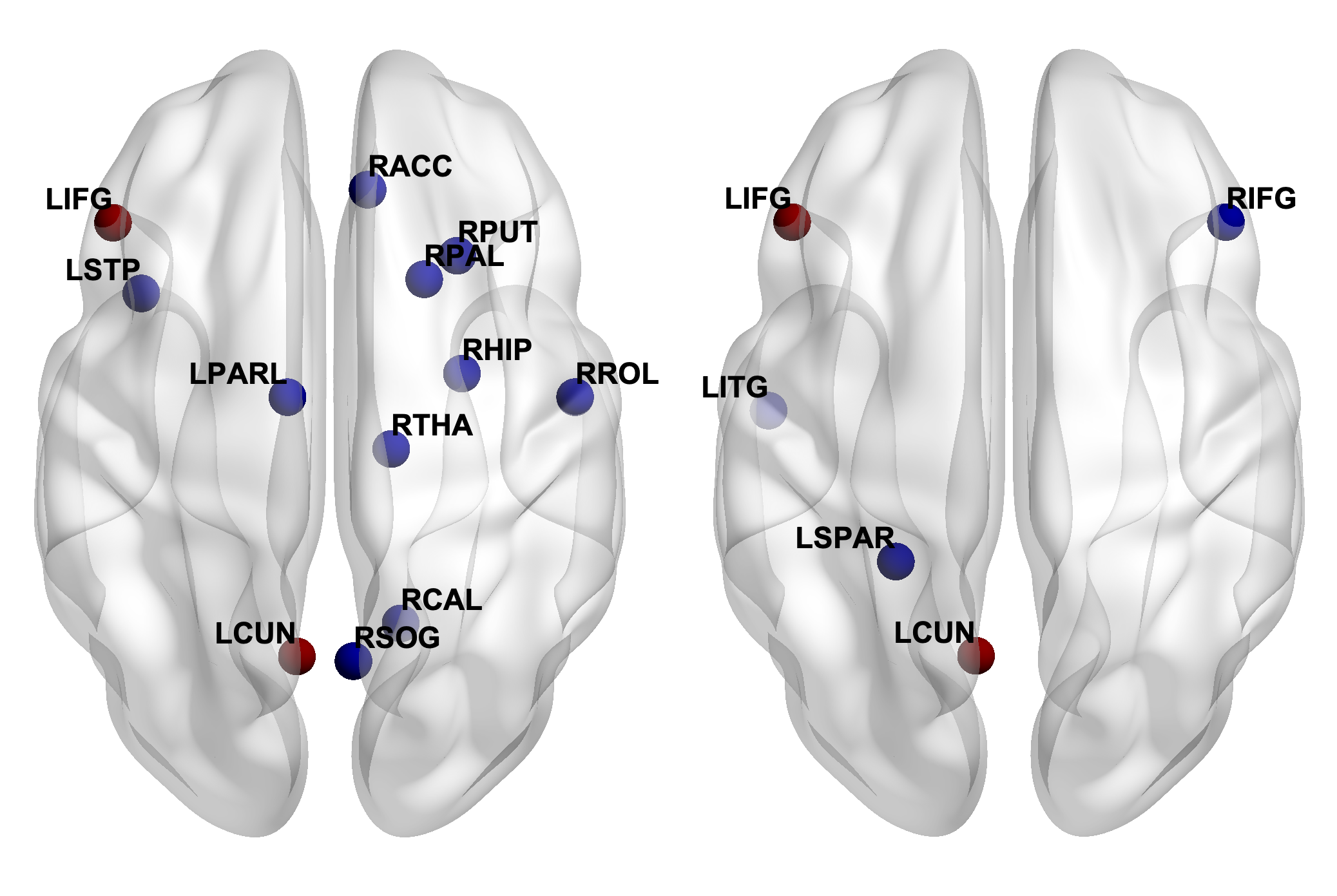


The efficiencies of these brain regions were significant covariates in predicting overall survival across cross-validation loops for the connectome (left) and gray matter volumes (right) models. Overlapping regions between the two models are shown in red. RACC: right anterior cingulate, RTHA: right thalamus, LIFG: left inferior frontal triangularis, RROL: right rolandic, LSTP: left superior temporal pole, RHIP: right hippocampus, RCAL: right calcarine, LCUN: left cuneus, RSOG: right superior occipital gyrus, LPARL: left paracentral lobule, RPUT: right putamen, RPAL: right pallidum, RIFG: right inferior frontal triangularis, LITG: left inferior temporal, LSPAR: left superior parietal. Figure created using BrainNet Viewer ^1^.

1 Xia, M., Wang, J. & He, Y. BrainNet Viewer: a network visualization tool for human brain connectomics. *PLoS One* **8**, e68910, doi:10.1371/journal.pone.0068910 (2013).
